# Book Notices

**Published:** 1895-07

**Authors:** 


### Book Notices.

INTERNATIONAL CLINICS: A Quarterly of Clinical Lectures on Medicine, Neurology, Surgery, Genito-Urinary Surgery, Gynecology, Obstetrics, Ophthalmology, Laryngology, Pharyngology, Rhinology, Otology, and Dermatology. By Professors and Lecturers in the leading Medical Colleges of the United States, Germany, France, Great Britain and Canada. Edited by Judson Daland, M. D. (Univ. of Penn.), Philadelphia, Instructor in Clinical Medicine and Lecturer on Physical Diagnosis in the University of Pennsylvania, etc., etc.; J. Mitchell Bruce, M. D., F. R. C. P., London, England, Physician to and Lecturer on the Principles and Practice of Medicine in the Charing Cross Hospital; David W. Findley, M. D., F. R. C. P., Aberdeen, Scotland, Professor of Practice of Medicine in the University of Aberdeen, Physician to, and Lecturer on Clinical Medicine in, the Aberdeen Royal Infirmary, etc. Volume I, Fifth Series. Price, per volume, cloth, $2.75; Leather, $3. J. B. Lippincott Company, Publishers, Philadelphia. 1895.

This volume contains three hundred and fifty-nine pages, fourteen plates, and forty-three figures. It is a report of forty-five clinical lectures delivered by professors and lecturers in the leading medical colleges of this and other countries. To those who are familiar with the "clinics," it is not necessary to say that these are not merely dry didactic lectures, but are from clinical work, and in each lecture the cases are introduced, and a concise history of the case is given, the symptoms are described, a diagnosis is made, and the treatment, either medicinal or operative, is applied.

The range of subjects discussed in this volume is a wide one, more than one hundred pages being devoted to medicine, sixty pages to neurology, seventy-four pages to surgery, twenty-four pages to genito-urinary and venereal diseases, forty-three pages to gynecology and obstetrics, eighteen pages to ophthalmology, nineteen pages to laryngology, pharyngology, rhinology and otology, and twelve pages to dermatology. H.

---

MATERIA MEDICA AND THERAPEUTICS, for Physicians and Students, by John B. Biddle, M. D., late Professor of Materia Medica and General Therapeutics in the Jefferson Medical College, Philadelphia. Thirteenth edition, revised, rearranged and enlarged, with special reference to Therapeutics, Toxicology, the Physiological Action of Medicine, and containing all the Preparations and Remedies described in the U. S. Pharmacopœia of 1890, to which the work has been made to conform. By Clement Biddle, M. D., Medical Corps U. S. Navy. With numerous illustrations. Price, cloth, $4.00; sheep, $5.00. Philadelphia. P. Blakiston, Son & Co., No. 1012 Walnut St., 1895.

This work was first issued in 1865, and the fact that it has passed through thirteen editions, attests its popularity and usefulness.

The present edition contains 714 pages. The work is divided into three parts.

Part first is contained in half dozen pages and treats of mechanical remedies—blood letting, setons, issues, etc.

Part second treats of imponderable remedies—light, heat, cold, electricity and massage.

Part third, constituting the bulk of the work, treats of pharmacological remedies.

The author divides these into four classes, and these classes are again divided into various orders.

Class 1 treats of neurotics, and includes those drugs whose chief physiological effect is exerted upon the nervous system. This class of drugs is divided into eight orders.

Eccritics, divided into six orders, constitutes the second class. Under this class come emetics, cathartics, diuretics, emmenagogues, etc.

Hæmatics, or those remedies chiefly affecting the blood, are treated in class 3, and are divided into three orders.

Class 4 treats of topical medicines.

The author gives prominence to medicines derived from the vegetable kingdom, and excellent illustrations are given of many of the medicine-bearing plants.

The appendix contains many valuable tables.

The clinical index is full.

The author's style is clear, and you are at no loss to understand him.

For the student and general practitioner this is an excellent book.

This last revision brings the work up to date. C. J. F.

---

PRACTICAL URANALYSIS AND URINARY DIAGNOSIS: A Manual for the Use of Physicians, Surgeons, and Students. By Charles W. Purdy, M. D., Queen's University; Fellow of the Royal College of Physicians and Surgeons, Kingston; Professor of Urology and Urinary Diagnosis at the Chicago Post-Graduate Medical School. Author of "Bright's Disease and Allied Affections of the Kidneys"; also of "Diabetes: Its Causes, Symptoms, and Treatment." With Numerous Illustrations, including Photo-Engravings and Colored Plates. In one Crown Octavo volume, 360 pages, in Extra Cloth, $2.50 net. Philadelphia: The F. A. Davis Co., Publishers, 1914 and 1916 Cherry Street.

This is quite a comprehensive work embracing not only the analysis of urine, but urinary diagnosis, the diseases of the urinary organs, and the condition of the urine in other diseases.

Sixty-six pages are devoted to the consideration of the composition of normal urine and the proper methods of examination. In this section each constituent has been considered, so far as at present known, in the following order: Its chemical nature and composition; its source in the economy; the significance of its increase or decrease in the urine, with the relations of these to metabolic processes, food supply, physical surroundings, and tendency toward disease; also the most approved methods of its detection and determination.

In dealing with abnormal urine, the chemical nature and composition of each morbid constituent, its source in the economy, the clinical significance of its appearance in the urine, and the best methods of detecting its presence and determining its quantity have been carefully described.

The second division of the book deals with Urinary Diagnosis, and as this section deals with the special features of the urine, indicating a pathological condition, together with a description of the leading clinical symptoms of each disease, it, like the preceding section, will be found of great practical value.

In addition to the other valuable features of this work, it contains, in the form of an appendix, a chapter of sixteen pages on examination of urine for life insurance. H.

